# Taking the Myc out of cancer: toward therapeutic strategies to directly inhibit c-Myc

**DOI:** 10.1186/s12943-020-01291-6

**Published:** 2021-01-04

**Authors:** Sarah K. Madden, Aline Dantas de Araujo, Mara Gerhardt, David P. Fairlie, Jody M. Mason

**Affiliations:** 1 Department of Biology & Biochemistry, University of Bath, Claverton Down, Bath, BA2 7AY UK; 2 Division of Chemistry and Structural Biology and ARC 1066 Centre of Excellence for Innovations in Peptide and Protein Science, Institute for Molecular Bioscience, The University of Queensland, Brisbane, QLD 4072 Australia

**Keywords:** Oncogene, Transcription, Leucine zipper, Peptide, Protein-protein interaction

## Abstract

c-Myc is a transcription factor that is constitutively and aberrantly expressed in over 70% of human cancers. Its direct inhibition has been shown to trigger rapid tumor regression in mice with only mild and fully reversible side effects, suggesting this to be a viable therapeutic strategy. Here we reassess the challenges of directly targeting c-Myc, evaluate lessons learned from current inhibitors, and explore how future strategies such as miniaturisation of Omomyc and targeting E-box binding could facilitate translation of c-Myc inhibitors into the clinic.

## Introduction to c-Myc and the Myc/Max/Mxd network

**Fig. 1:**
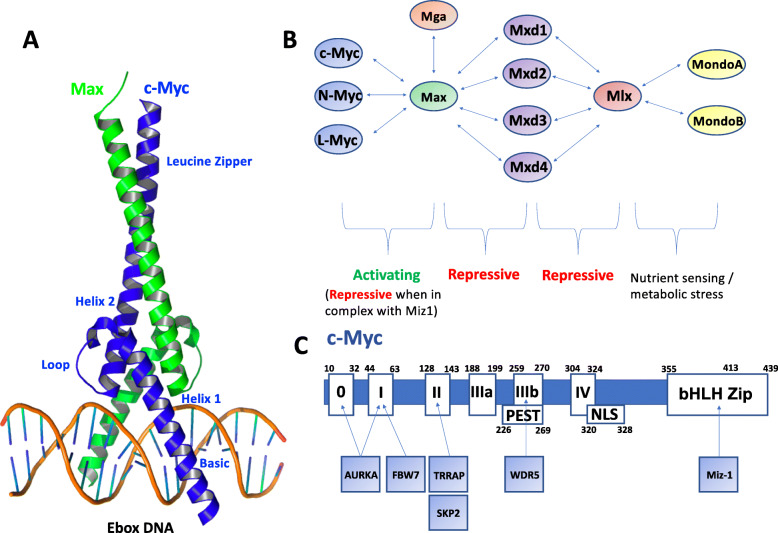
The structure and interaction partners of c-Myc. **a** Crystal structure of the c-Myc/Max dimer bound to E-box DNA (PDB ID 1NKP) [2]. **b** Scheme showing activating and repressing interaction partners, represented by double headed arrows, within the Myc/Max/Mxd network [3, 4]. **c** Different regions of c-Myc protein, including the MYC boxes (0, I, II, IIIa, IIIb, IV), Nuclear Localization Sequence (NLS) and bHLHZip domain along with the binding sites of its key interaction partners [5–10]

c-Myc is a transcription factor that belongs to the basic-helix-loop-helix-leucine zipper (bHLHZip – Fig. 1a) family present in the cell nucleus, where it acts to regulate cell growth, differentiation, metabolism and death, and is frequently dysregulated in many human cancers [1]. It is the prototype member of the Myc family that also encompasses N-Myc and L-Myc proteins in mammalian cells, all of which are highly homologous but distributed differently. c-Myc is ubiquitous and highly abundant in proliferating cells, whereas N-Myc and L-Myc display more restricted expression at distinct stages of cell and tissue development.

Myc proteins exist within the Myc/Max/Mxd network (Fig. 1b). To fold and become transcriptionally active c-Myc must first heterodimerize with Max, a process governed by the coiling of their bHLHZip domains. Once dimerized, the c-Myc/Max complex acts as a master transcriptional regulator by binding via its basic region to a specific DNA consensus sequence CANNTG, known as the Enhancer-box (E-box) (Fig. 1a). Within the network, c-Myc can only heterodimerize with Max, whereas Max is more promiscuous and able to homodimerize or heterodimerize with other factors that share a bHLHZip motif. These include proteins of the Mxd family (Mxd1-Mxd4, formally called Mad proteins) as well as Mnt (a protein distantly related to Mxd-family), and the much larger Mga, an unusual protein that contains both a bHLHZip motif and a T-domain DNA-binding motif [11, 12]. These additional Max associations are proposed to function as antagonists of the Myc family. They compete effectively for interactions with Max and attenuate c-Myc/Max activity by obstructing E-box binding sites, thereby functioning as transcriptional repressor complexes [13, 14]. Additionally, Max/Mxd and Max/Mnt are able to actively repress gene transcription by recruiting co-repressors Sin3 and histone deacetylase (HDAC), tagging histones for epigenetic repression [15]. The MLX/MONDO system (Fig. 1b) operates in parallel to the Myc/Max/Mxd network and is involved in regulating metabolism [3]. Such proteins are mainly present in the cytoplasm and can shuttle to different cell compartments in response to glucose and other metabolic stimuli.

## Myc interaction partners

c-Myc is a 62 kDa protein of 439 residues and comprises a series of functional domains which facilitate interactions with a diverse range of binding partners, each of which has distinctive roles in the c-Myc interactome (Fig. 1c). The N-terminal region contains a transcription transactivation domain (TAD), and three highly conserved MYC boxes (MB0, MBI, MBII) involved in transcriptional regulation and protein degradation. Located centrally is a PEST domain (rich in proline, glutamate, serine and threonine), a nuclear localization sequence (NLS), and three other conserved MYC boxes (MBIIIa, MBIIIb, and MBIV) that are implicated in transforming activity, transcription and apoptosis. The C-terminal region is approximately 100 residues long and comprises the (bHLHZip) domain responsible for DNA binding on promoters of specific target genes and for dimerization with obligate partner, Max (Fig. 1a) [16–20].

The binding of the c-Myc/Max dimer to E-box DNA can activate gene transcription through the recruitment of the transformation/transcription domain-associated protein (TRRAP), recruiting the histone acetytransferase complexes TIPS60 and GCN5, p300/CBP-associated factor and ATP-binding protein TIP48 to the vicinity of E-box sites. This leads to acetylation of histones H3 and H4, thereby opening chromatin structure and allowing RNA polymerase II machinery to access the core promoter, ultimately leading to transcription [13]. c-Myc also regulates elongation pause release through interaction with elongation factor and kinase P-TEFb, which in turn phosphorylates the C-terminus of RNA polymerase II and the release of negative elongation factors [21]. Interaction of c-Myc with additional cofactors such as WDR5, PAF1 (RNA polymerase II-associated factor 1 homologue) and bromodomain protein BRD4, are also likely to play important roles in stabilizing or directing the interaction of c-Myc to certain chromatin locations (Fig. 1c) [22, 23]. High levels of multiple numbers of these different factors are thought to lead to ‘super-enhancers’ that enable the collective target genes to become highly expressed.

c-Myc can also repress transcription, although this is less well-characterized than its ability to activate transcription. Repression of certain target genes has been correlated with Myc antagonising transcription factor Miz-1 (Myc-interacting zinc finger protein 1), which binds via the HLH regions within c-Myc (Fig. 1c) [24]. This association accounts for less than 40% of Myc-repressed genes, suggesting other interactors may contribute to gene repression. The c-Myc interactome is large and complex and additional c-Myc interacting proteins are continually being profiled with new screening tools such as BioID, an in-cell biotin-labelling mass spectrometry method for mapping of local protein-protein interaction networks. This technique has recently been used to identify additional factors such as the G9a H3K9-methyltransferase complex that interacts with c-Myc to control transcriptional repression at MBII sites, thereby revealing another c-Myc repression pathway that was independent of Miz-1 interaction [25, 26]. In another BioID study, the Protein Phosphatase-1 (PP1)/Protein Phosphatase-1 Nuclear-targeting Subunit (PNUTS) phosphatase complex was found to modulate c-Myc binding to chromatin through dephosphorylation [27].

c-Myc regulates many cellular processes through targeting as many as 15% of all genes [28]. Target genes include those involved in cell cycle regulation, such as cyclins D1, D2, B1 and cyclin-dependent kinase 4 (CDK4), and c-Myc also decreases and interferes with the function of p21 and p27 inhibitors of CDK. Metabolism is regulated by c-Myc through enolase A, hexokinase II, lactate dehydrogenase A, phosphofructokinase, and glucose transporter I. Other target genes include those involved in protein synthesis, ribosome biogenesis and cell adhesion [28].

c-Myc is tightly regulated in the cell by a range of upstream and downstream mechanisms at the genetic, mRNA and protein level, which can become disrupted in cancer cells. For example, in response to phosphorylation events at the TAD region, c-Myc interacts with ubiquitin ligases FBW7 and SKP2 at MBI and MBII respectively, which promote its proteasomal degradation reducing its half-life to 15–20 mins in the absence of other signals (Fig. 1c) [29–31]. Association with the mitosis checkpoint protein, Aurora kinase A (AURKA) at MB0 and MBI regions acts to stabilize c-Myc by inhibiting its interaction with FBW7 [32]. Interestingly, some regulators of c-Myc degradation are encoded by c-Myc target genes. c-Myc can also induce apoptosis to provide an additional level of control against unrestrained cell growth [5, 33].

## c-Myc as a cancer target

Expression of c-Myc is tightly controlled in normal cells, but becomes dysregulated and overexpressed in most human cancers [34], making it one of the most important human oncogenes. This can be driven by many mechanisms at the DNA, RNA and protein level, although rarely through direct c-Myc mutation [34–36]. Overexpression of c-Myc can increase interaction with lower affinity E-boxes, triggering tumorigenesis by changes in gene activation such as those regulating cell proliferation and growth that would not occur at normal physiological concentrations [35, 37–40].

Over the years, inhibition of the Myc family has been modelled in vivo using genetic knockout, siRNA or indirect mechanisms that impede c-Myc function. Lipid nanoparticle-based formulations (DCR-MYC) have been used to deliver siRNA into tumor cells, leading to inhibition of translation and expression of the c-Myc protein [41]. This approach was later found to not meet therapeutic expectations and was halted, however work using antisense oligonucleotides to target c-Myc mRNA continues [42]. More recently, the inhibitor Omomyc, a c-Myc dominant negative protein [43–45], has shed light on the impact of directly inhibiting c-Myc-mediated malignancy. Omomyc has been shown to induce significant tumor regression in a range of cancers, even those in which c-Myc is not the driver oncogene, thereby validating c-Myc as a potential drug target in cancer. The ubiquitous nature of c-Myc deregulation in cancer also makes its inhibition an attractive treatment option for the many cancers where there are few treatment options and/or there is a poor prognosis. This list includes cancers of the lung, pancreas, oesophagus and brain amongst others [46]. For example, c-Myc features prominently in pancreatic cancer where only 5% of patients survive for more than 5 years [47].

## Therapeutics that bind to c-Myc are likely to target other Myc homologues

Although c-Myc is a key therapeutic target, it may also be advantageous to simultaneously target the homologous N-Myc and L-Myc proteins that have been shown to be involved in tumor maintenance and progression [34, 48–50]. N-Myc expression is elevated due to amplification of the *MYCN* gene in many cancers [51]. In neuroblastoma, this amplification is found in 50% of high-risk neuroblastomas and the amplification is believed to drive cancer initiation rather than progression [52]. Around 20% of neuroendocrine Small Cell Lung Cancers (SCLCs) are associated with amplification of *MYCN, MYC or MYCL* genes [53]. Fortunately, the high sequence and structural similarity of these proteins should facilitate the development of pan-Myc-selective inhibitors.

## c-Myc as a therapeutic target in other diseases

c-Myc is a master regulator of immunometabolism and its dysregulation is implicated in inflammatory, autoimmune, metabolic and other non-cancerous disorders, although it remains poorly understood. The lack of an effective inhibitor that directly targets c-Myc compromises studies investigating the potential of c-Myc inhibition as a therapeutic strategy to treat chronic diseases. Nevertheless, recent reports using indirect inhibitors or transgenic mice have shown some potential. It was recently verified that c-Myc expression is upregulated in group 2 innate lymphoid cells (ILC2s) in the blood of asthma patients. Using a mouse model of allergic inflammation, it was found that inhibition of c-Myc repressed ILC2 activity, causing reduction in airways inflammation and other pathogenic responses [54]. These findings suggest that targeting c-Myc may unlock novel strategies to combat asthma. As recently reviewed [55], c-Myc upregulation has also been shown to be a hallmark of dysregulated cystoproteins (Polycystin-1 and -2). c-Myc is strongly linked to renal cystic diseases and onset of polycystic kidney disease (PKD) in animal models, suggesting the potential for c-Myc inhibition in PKD treatment. In the spectrum of inflammatory illnesses, c-Myc dysfunction has been reported in patients with Crohn’s disease [56] and other chronic gastrointestinal disorders [57]. In a model of autoimmune encephalomyelitis, treatment with c-Myc inhibitor 10058-F4 suppressed the ability of Th1-differentiated T-cells to induce inflammation [58]. Low expression of c-Myc in haploinsufficient mice has been shown to be responsible for extended lifespan, resistance to many age-associated pathologies, higher metabolic rate and healthier lipid metabolism, suggesting an important role for c-Myc in regulating aging and lifespan [59]. Together, these preliminary reports support a pressing need for more potent and effective inhibitors of c-Myc activity to unravel the important roles of this protein in physiological homeostasis and the undesirable consequences of its aberrant expression in disease.

## Challenges and considerations in targeting c-Myc

Initially c-Myc was regarded as a risky therapeutic target, due to the possible serious side effects resulting from inactivating a master regulator protein considered essential for normal cell survival and proliferation [60]. This concern was supported by studies where c-Myc germline knockout mice were generated using a gene-targeted transgene approach, and found to be lethal in homozygotes at 10.5 days gestation [61]. However, more recent mouse studies have shown that Myc family inhibition, triggered by a genetically expressed modulator (Omomyc), had little to no side effects in normal tissue [45]. Although significant side-effects were observed in regenerating tissues, such as skin, these were found to be well-tolerated even over prolonged timeframes and rapidly reversed after cessation of inhibitor expression.

Despite extensive evidence of the critical role of c-Myc in many cancers, the viability of directly targeting c-Myc remains uncertain [34, 44, 45, 60]. Ideally, aberrant transcription could be halted by targeting c-Myc with drugs that impede its dimerization with Max and hence DNA binding. However, the disordered nature of unbound c-Myc significantly complicates drug development. c-Myc is an Intrinsically Disordered Protein (IDP), and therefore its extended unstructured surface particularly in the unbound bHLHZip domain lacks the requisite “hotspots” and deep hydrophobic pockets that are typically targeted effectively using conventional small molecule drugs. Small molecule modulators may also not selectively bind c-Myc over the many bHLHZip motifs found in other transcription factors. Further, the location of endogenous c-Myc in the nucleus presents another limitation on therapeutics, which are required to both penetrate cells and translocate efficiently to the nucleus. Despite substantial efforts by the pharmaceutical sector to date, only a few compounds have been reported to interfere directly with c-Myc/Max/DNA complexation in vivo, highlighting the difficulty in developing potent and selective c-Myc inhibitors as drug leads. Small molecule inhibitors have been identified with affinity at nanomolar concentrations [62]. The interaction of c-Myc with Miz-1 has been shown to suppress tumors, but is also involved in oncogenic transformation [63–65]. Therefore, the development of therapeutics that could modulate this interaction requires careful consideration.

There are many lines of inquiry described elsewhere to derive molecules that indirectly modulate c-Myc by inhibiting the activity of the many upstream and downstream proteins that interact with and impact upon its activity, or that target it for degradation. For example, approaches to target c-Myc indirectly, by binding BRD4, CDK7/9, or G-quadruplex DNA, have been reported and are reviewed elsewhere [13, 48, 66–69]. Here we focus on the more desirable but challenging task of inhibiting c-Myc directly.

Recent work is beginning to demonstrate that IDPs can form liquid droplets in the cell. These are sometimes referred to as “membrane-less organelles”, due to a liquid-liquid phase transition induced by IDP-IDP interactions [70–72]. Implications of liquid droplets in therapeutic targeting of IDPs are not yet known, but it is reasonable to assume that their formation may reduce inhibitor efficacy due to poor diffusion into the droplets and/or poor accessibility to the target protein within.

## Current strategies for directly targeting c-Myc

### Small molecule inhibitors

**Fig. 2:**
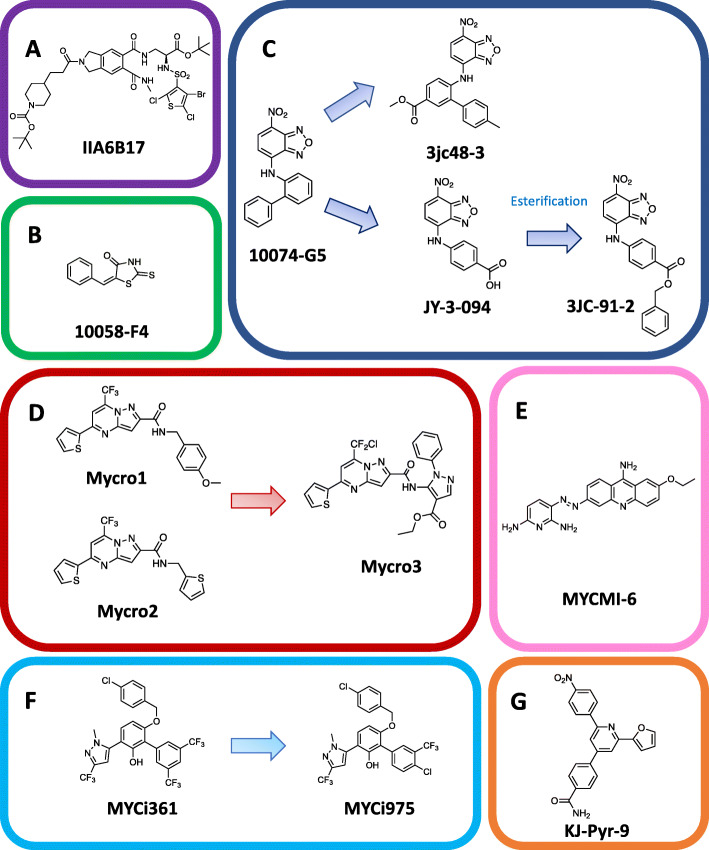
Small molecule inhibitors of c-Myc. **a** IIA6B17 [73]. **b** 10058-F4 [76]. **c** Evolution of 10074-G5 [85]. **d** Evolution of Mycro1 and Mycro2 [88]. **e** MYCMI-6 [91]. **f** Evolution of MYCi [94]. **g** KJ-Pyr-9 [62]

To date, no small molecule inhibitors that directly target the c-Myc/Max interaction have progressed to clinical trials. This is likely due to issues with target selectivity, rapid metabolism and low potency, as discussed above. However, a number of conventional small molecules have been identified to inhibit c-Myc/Max dimerization or DNA binding (Table 1). The IDP structure of the c-Myc and Max monomers has impaired discovery of novel modulators using traditional structure-based design, leading to inhibitors being identified mostly by high throughput screening of chemical libraries [73–76, 87, 91, 125]. Initial small molecule library screens identified compounds IIA6B17 (Fig. 2a) and NY2267 [73, 75] as capable of interfering with c-Myc/Max interaction. However, both compounds were later found to have poor selectivity and also acted upon c-Jun, most likely due to similarities in the leucine zipper components [126]. This lack of specificity is a common problem for small molecule inhibitors of c-Myc function.

**Table 1:** Properties of small molecules, peptides and proteins that inhibit Myc activity

Inhibitor type	Inhibitor	Mechanism of action (A, B or C)^a^	Inhibitor of Myc/Max binding to DNA in vitro	Activity in vitro	Reduction of cancer cell growth/proliferation	Tumor reduction in animal models	Reference
Small Molecules	IIA6B17	B	Yes	**IC**_**50**_ **=** 50 ± 25 μM (EMSA, inhibition of E-box binding)	Yes	–	[73, 74]
	NY2267	B	Yes	**IC**_**50**_ **=** 36.5 μM (EMSA, inhibition of E-box binding)	–	–	[75]
	10,058-F4	B	Yes	***K***_**D**_ **=** 42 μM (Binding to c-Myc) 15 μM (SPR)	Yes	No	[76–81]
	10,074-G5	B	Yes	**IC**_**50**_ **=** 146.8 μM (EMSA, inhibition of E-box binding) ***K***_**D**_ **=** 20 μM (Binding to c-Myc) 18 μM (SPR)	Yes	No	[76, 77, 82, 83]
	JY-3-094	B	Yes	**IC**_**50**_ **=** 33 μM (EMSA, inhibition of E-box binding)	Yes	–	[84, 85]
	3jc48–3	B	Yes	**IC**_**50**_ **=** 34.8 μM (EMSA, inhibition of E-box binding)	Yes	–	[86]
	Mycro1, Mycro2	B	Yes	**IC**_**50**_ **=** 30 ± 5 μM (Mycro1) and 23 ± 4 μM (EMSA, inhibition of dimerization and E-box binding)	Yes	–	[87, 88]
	Mycro3	B	Yes	**IC**_**50**_ **=** 40 ± 13 μM (FP competition assay, inhibition of E-box binding and dimerization)	–	Yes	[88–90]
	MYCMI-6	B	–	***K***_**D**_ = 1.6 ± 0.5 μM (SPR, binding to c-Myc)	Yes	Yes	[91]
	KJ-Pyr-9	B	Yes	***K***_**D**_ = 6.5 ± 1.0 nM (Backscattering Interferometry, binding to c-Myc)	Yes	–	[62, 92, 93]
	MYCi361	B, C	Yes	***K***_**D**_ = 3.2 μM (FP competition assay, binding to c-Myc)	Yes	Yes	[94]
	MYCi975	B, C	–	***K***_**D**_ = 2.5 μM (FP competition assay, binding to c-Myc)	Yes	Yes	[94]
	Celastrol and analogues	A,C	Yes	**IC**_**50**_ **=** 67 ± 2 μM (Celastrol) (EMSA, inhibition of E-box binding)	Yes	Yes ^b^	[95]
	JKY-2-169	A	Yes	**IC**_**50**_ **=** 11.6 ± 2.3 μM (EMSA, inhibition of E-box binding)	–	–	[94, 96, 97]
	EN4	B	Yes	**IC**_**50**_ **=** 6.7 ± 2.3 μM (inhibition of E-box binding)	Yes	Yes	[98]
(Poly)peptide/mini-protein	Omomyc	A, B, C	Yes	***K***_**D**_ of Omomyc homodimer = nM range (Circular Dichroism spectroscopy, binding to E-box)	Yes	Yes	[43–45, 48, 49, 99–105]
	Max bHLHZ	A, B	–	–	Yes	–	[5]
	Mad1	A, B	Yes	–	Yes	–	[106]
	ME47	A	Yes	***K***_**D**_ = 15.3 ± 1.6 nM (EMSA, binding to E-box)	Yes ^c^	Yes ^c^	[107–110]
	Monoclonal antibody	B	Yes	–	–	–	[111]
	H1 peptide	B	Yes	–	Yes	Yes	[112–117]
	aMax/aMip	B	Yes	***K***_**D**_ = 460 μM (aMax), 250 μM (aMip) (Thermal denaturation monitored by CD, binding c-Myc in absence of DNA)	–	–	[118–120]
	Linked basic regions	A	–	–	–	–	[121–123]

^a^ Mechanisms of action: A) E-box inhibitor, B) Inhibitor of c-Myc/Max binding, C) c-Myc degradation promoter

^b^ Shown to inhibit tumor growth but likely due to another mechanism [124]

^c^ Transgene, not as a peptide alone

*-* indicates that this is currently unknown

Compounds 10058-F4 (Fig. 2b) and 10074-G5 (Fig. 2c) were identified from a library of 10,000 compounds using a yeast two-hybrid screen and shown to bind to c-Myc in an IDP state, preventing it from adopting the conformation necessary for dimerization with Max [76]. Thioxothiazolidinone 10058-F4 was found to bind to Helix 2 and the c-Myc leucine zipper with a modest affinity with a ***K***_**D**_ of 42 μM; whereas 10074-G5 bound to Helix 1 and the basic region with a ***K***_**D**_ of 20 μM [48, 77]. To improve inhibitor potency, 10058-F4 and 10074-G5 were covalently linked to create a bitopic inhibitor, leading to a modest improvement in binding affinity [127]. A later study also sought to optimize 10058-F4 potency through functional group modification, leading to improved inhibition of the growth of c-Myc-expressing cells [78]. In vitro, 10058-F4 inhibited pancreatic ductal adenocarcinoma, acute myeloid leukaemia and ovarian carcinoma, inducing a range of effects from apoptosis to cell cycle arrest [79–81, 128], but failed to reduce growth of human prostate cancer xenografts in mice [129]. This was attributed to its rapid clearance from mouse plasma due to low metabolic stability.

Studies have also been conducted to improve the potency of 10074-G5. Using EMSA experiments, the variant 3jc48–3 displayed four times greater potency than the parent molecule (Fig. 2c; Table 1) [86]. 10074-G5 was later redesigned to compound JY-3-094, which was a stronger inhibitor of c-Myc/Max dimerization [84, 85]. An esterified prodrug form of JY-3-094 (Fig. 2c), which masked the negative charge of the carboxylate and improved cell penetration, had reduced activity in vitro. Like 10058-F4, 10074-G5 has also been shown to be rapidly metabolized and poorly distributed in tumors in vivo despite promising in vitro potency [82].

Later small molecules, such as Mycro3 and MYCMI-6, have shown more promising activity in vivo (Fig. 2d and e) [89–91]. Mycro3 was developed from a pyrazolo [1,5-a] pyrimidine library based on two prototype c-Myc/Max inhibitors, Mycro1 and Mycro2, previously also identified using a high-throughput screening approach [87, 88]. In addition to improved pharmacokinetic properties relative to other c-Myc inhibitors, Mycro3 was shown to prolong survival and reduce tumor size in a KRas-driven pancreatic ductal adenocarcinoma mouse model [89].

Using a bimolecular fluorescence complementation cell-based assay to survey a 1990-compound library, a new small molecule inhibitor, MYCMI-6 (Fig. 2e), was found to be capable of selectively binding to the Myc family bHLHZip domain at low micromolar concentrations [91]. MYCMI-6 inhibited the c-Myc/Max interaction in cells and supressed tumor growth in several cancer cell lines, particularly those expressing high c-Myc protein levels, without cytotoxicity to normal human cells. Furthermore, MYCMI-6 promoted significant apoptosis and reduction of tumor proliferation in a neuroblastoma xenograft model in vivo.

In a stepwise screening approach for the high throughput identification of inhibitors with favorable pharmacokinetic and pharmacodynamic properties, a pharmacophore-based in silico screen of a 16 million compound library was used to identify hits with favorable drug-like properties. These hits were subjected to a secondary screen where c-Myc inhibition activity was assessed in vitro and in cells and later coupled to a rapid in vivo screen in mice bearing a c-Myc-dependent E-box luciferase reporter [94]. This led to identification of inhibitors MYCi361 and MYCi975 (Fig. 2f) that showed significant anti-tumor activity in mice and promising pharmacokinetic properties, such as high plasma concentration, longer half-life and improved tumor penetration. Small molecules such as MYCMI-6, MYCi361/975 and KJ-Pyr-9 show promise for further development as potential clinical candidates [62, 91, 94].

Among published inhibitors, KJ-Pyr-9 exhibited the highest reported binding affinity for c-Myc with ***K***_**D**_ of 6.5 nM (Fig. 2g, Table 1) [62]. KJ-Pyr-9 was isolated from a Kröhnke pyridine library where fluorescence polarisation (FP) was used to screen for c-Myc/Max dimerization inhibitors [130]. KJ-Pyr-9 has also demonstrated anti-cancer activity in vivo, inhibiting tumor growth in a human triple-negative breast cancer xenograft model with no acute toxicity.

Other studies have investigated alternative approaches towards identifying inhibitors of c-Myc. For example, in addition to identifying inhibitors of c-Myc/Max dimerization, small molecule inhibitors of the c-Myc/E-box DNA interaction have been identified. One example, celastrol is a naturally occurring compound that can bind c-Myc/Max to abrogate E-box binding [95, 131–134]. The α-helix mimetic, JKY-2-169, has also been shown to perturb the c-Myc/Max structure, thereby inhibiting the ability of the heterodimer to bind DNA [96]. Other peptidomimetics have been used to create small molecules of high potency for the c-Myc Helix 1 region [135], while certain other small molecules have been shown to stabilize Max homodimers [14, 136].

### Protein and peptide inhibitors

Synthetic proteins, peptides and mimetics now offer new opportunities to turn IDPs involved in pathology into tractable therapeutic targets (Table 1). This is because relative to small molecules, peptides can make multiple and diverse interactions with biological targets, including on expanded but shallow surfaces, enabling high affinity yet selective binding to protein-protein interfaces [137, 138]. Conversely, peptide drug candidates have long been associated with poor pharmacokinetic properties and traditionally ignored, in favor of more drug-like small molecules. Limitations include rapid proteolytic degradation, low membrane and cell permeability, low oral bioavailability, high clearance, and poor tissue distribution. However, peptide- and protein-based therapeutics have greatly increased in the last two decades with the advance of recombinant and synthetic chemical methods, allowing fast access to diverse peptides. A range of chemical strategies have been implemented to improve pharmacokinetic (PK) properties of peptides and proteins. For example, conjugation of certain moieties (lipids, PEGs, biopolymers) can extend their circulation lifetime in vivo and has led to successful clinical outcomes (e.g. liraglutide, insulin detemir, pegfilgrastim) [139–141]. Peptides can also be designed to impart membrane permeability via conjugation with cell- and brain-penetrating sequences [142, 143]. Modifications of the peptide backbone, such as N-methylation or incorporation of D-amino acids or cyclization approaches, can impede proteolysis [138, 144–147]. Some recent peptide stapling strategies have been shown to stabilize flexible peptide epitopes to create robust cyclic structures that can bind biological targets with high affinity, resist proteolytic degradation, and permeate cells more efficiently. For example it has been reported that hydrocarbon stapling can generate improvement in proteolytic stability both by increasing α-helicity and by inhibiting proteolysis at cleavage sites [146, 148]. In the next section we review peptides that have been used to target c-Myc.

## Omomyc

### Omomyc as an inhibitor of c-Myc

**Fig. 3:**
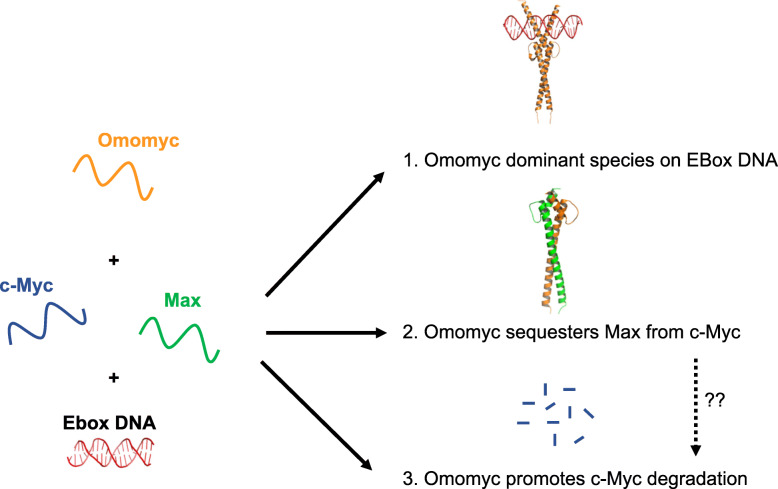
Proposed mechanisms of Omomyc. Studies have suggested that: 1. Omomyc (Orange) homodimer blocks c-Myc/Max dimer from binding to E-box DNA (Red). 2. Omomyc binds to Max (Green), sequestering Max from c-Myc (Blue) and 3. In the presence of Omomyc, c-Myc levels are reduced due to proteasomal degradation, a process potentially tiggered by 2 [49, 105].

Omomyc is a 91-residue c-Myc dominant negative mini-protein developed by Soucek et al. and is the most extensively studied peptide-based c-Myc inhibitor to date [99] (Fig. 3). Omomyc contains four amino acid substitutions in the leucine zipper domain of c-Myc (E410T, E417I, R423Q, R424N), designed using molecular modelling to remove electrostatic clashes that impede c-Myc dimerization, thus allowing Omomyc homodimerization as well as heterodimerization with c-Myc and Max. It was predicted that Omomyc had the potential to disrupt the Myc/Max/Mxd network and act as an inhibitor of c-Myc function [99], and it has been shown to induce tumor regression in multiple cancer models, including pancreatic, lung, breast and brain cancer through a range of effects including reduced cell proliferation and increased apoptosis [43–45, 49, 100–104, 149]. Transcriptomic analysis revealed several genes downregulated by Omomyc treatment and downstream effectors of dysfunctional c-Myc transformation [149, 150]. Although mostly explored as a transgenic expressed vector in cells, the anticancer profile of Omomyc played an important part in establishing the therapeutic potential of c-Myc inhibition.

### Disentangling the mechanism of Omomyc

The exquisite bHLHZip chimera of Omomyc allows for selective binding within the Myc/Max/Mxd network and a mode of action that is different from complete loss-of-function obtained by gene knockout or RNA interference [103]. In co-immunoprecipitation experiments, using various cell lines where Omomyc is ectopically expressed or exogenously applied, Omomyc was shown to interact with Max, c-Myc and other Myc members, itself to form a homodimer, and with Miz-1, but not with members of the Mxd family or other factors such as HIF-1α [103] [105]. Omomyc may therefore act via multiple inhibitory mechanisms, such as sequestering c-Myc or Max away from c-Myc/Max dimerization or by directly interfering with E-box DNA complexation of transcription factors (Fig. 3).

Accumulated evidence over the years strongly supports Omomyc inhibiting c-Myc function by directly blocking E-box binding sites, thus preventing c-Myc/Max dimer from binding to promoter regions of c-Myc target genes [49, 99, 105, 151]. Omomyc forms a well-defined homodimer that complexes to DNA in a similar manner to the c-Myc/Max dimer but with likely greater thermodynamic stability than c-Myc/Max according to crystal structure analysis [105]. In vitro, Omomyc homodimers bound E-box DNA with higher affinity than c-Myc/Max or Omomyc/c-Myc dimers and Max homodimers based on gel shift assay data [105]. In cells, chromatin immunoprecipitation (CHIP) experiments revealed that Omomyc treatment remarkably reduced c-Myc binding to E-box sites of target genes. Further, the ability of Omomyc to repress c-Myc-dependent transcription was significantly attenuated by mutating DNA-binding residues located at the basic region, suggesting that promoter-binding plays an important role in Omomyc action [105]. Also, using CHIP technology, it was verified that Omomyc can bind to DNA in cells as either a homodimer or a heterodimer with Max, but not as a heterodimer with c-Myc [104]. The inability of Omomyc/c-Myc heterodimers to bind DNA was also observed by Circular Dichroism (CD) spectroscopy [49]. It was further verified that Omomyc can outcompete other Max dimerization partners, such as Mxi1, MGA, Mnt and Mxd3, away from immobilized E-box-DNA beads in a proteomic pulldown assay using Ramos cell lysates [104]. Others observed that Omomyc discriminates between different classes of promoters, showing a stronger repressive effect on unoccupied cognate sites that become invaded by rising c-Myc levels during tumorigenesis [105]. This observation offers a potential explanation for why Omomyc has been shown to have low toxicity as essential c-Myc target genes are still expressed.

Another proposed mechanism for Omomyc activity that is independent of DNA binding is the sequestration of c-Myc and Max into non-transcriptional heterodimeric complexes with Omomyc to rebalance c-Myc/Max ratio to non-oncogenic levels. This is supported by observations that c-Myc and Max co-immunoprecipitated with Omomyc in cells. However, the binding between Omomyc, c-Myc and Max has not been determined in the absence of DNA, and it is unclear how effectively Omomyc heterodimers can outcompete Omomyc/Omomyc or c-Myc/Max dimerization under a DNA-free scenario. To date, interaction of c-Myc and Max with Omomyc has been observed only at high micromolar concentrations through NMR structure analysis [49], whereas CD spectra and thermal denaturation curves indicated that, without DNA, Omomyc binds to itself and Max with comparable affinity.

Recently it has been proposed that Omomyc interference with c-Myc/Max binding could produce excessive free c-Myc monomer in cancer cells for ubiquitination and proteasomal degradation [104]. Omomyc treatment was shown to reduce c-Myc levels in HCT116 colon cancer cells and Ramos lymphoma cells. This effect could be significantly attenuated upon co-incubation with the proteasome inhibitor MG-132, implying a role for proteasomal degradation of c-Myc protein in restoring c-Myc levels under Omomyc influence. Treatment with a different c-Myc inhibitor, MYCi361, also reduced c-Myc stability [94]. This destabilization effect correlated with an increase in c-Myc T58 phosphorylation by glycogen synthase kinase 3 beta (GSK3b).

### Omomyc in the clinic?

In addition to the importance of Omomyc-expressing transgenic mouse studies in establishing that c-Myc inhibition is therapeutically relevant, these studies also suggest that Omomyc, or an Omomyc-derived molecule, could be used as a therapeutic themselves [44, 45, 49, 101, 152]. Clinical trials using Omomyc and variants are planned by Peptomyc S.L. and expected to commence in 2021 [12]. Consideration should also be given not only to whether Omomyc can be translated to the clinic, but how Omomyc treatment might be used in a clinical setting. Work has demonstrated that intermittent expression of Omomyc in KRas-driven lung cancer was able to trigger rapid regression of tumors, suggesting that Omomyc could be given in short bursts at regular intervals in a clinical setting [44, 151]. It was also shown that Omomyc was superior to paclitaxel in reducing tumor growth in a xenograft mouse model of human H1975 cells [49]. Combination therapy of Omomyc and paclitaxel was more effective than either drug alone, demonstrating a possible route for Omomyc to the clinic.

### Challenges to Omomyc as a therapeutic protein

Omomyc size and structure may provide a challenge to its use in the clinic [12, 45, 49]. However, studies have demonstrated that despite its size, Omomyc can penetrate into cells, with the basic region potentially acting as an internal protein transduction domain (PTD), most likely through an ATP-dependent uptake mechanism [49, 104]. Omomyc shares similar cell uptake pathways as other highly positively charged cell-penetrating peptides, which are cell-type dependent but mostly utilize clathrin-mediated endocytosis and macropinocytosis mechanisms [49]. This latter route is advantageous for tumor treatment, as cancer cells are sensitized to macropinocytosis due to accelerated metabolism [153]. Moreover, some Omomyc was also detected in the nucleus in a range of cell lines, with anticancer activity in several types of cancer cells and lung cancer animal models [49, 104]. Omomyc uptake into cells was also aided by attaching a functional penetrating ‘Phylomer’ peptide (FPPa), enabling Omomyc to inhibit tumor growth in triple negative breast cancer using an allograft model [149]. An Omomyc fusion protein, Omomyc-FN-H6, was able to be delivered into cells in bacterial inclusion bodies, and was shown to induce cytotoxic effects in a triple negative breast cancer cell mouse model [154]. Another proposed strategy to increase Omomyc antitumor efficacy is to introduce modifications that impair binding to Miz-1, so that deactivation of cell proliferation and growth regulators programmed by this repressive pathway can be halted. For instance, Omomyc expression in 293 T cells was shown to repress activation of cell cycle inhibitor CDKN1A via interaction with Miz-1 [103].

Treatment with low micromolar concentrations of the ~ 10 kDa Omomyc protein has led to significant antiproliferative responses in cancer cells with amplified c-Myc levels (lymphoma, colon and lung cancer), promoting c-Myc transcriptional shutdown in a similar fashion as described for its transgenic vector. More recently, the preclinical efficacy of Omomyc intranasal administration was evaluated in a mouse model of lung adenocarcinoma. After four weeks of treatment, Omomyc halted tumor progression, whereas the tumor doubled in volume with vehicle, and promoted recruitment of T cells to the tumor site. Omomyc was also tested intravenously, although at high doses, and shown to be superior to paclitaxel in reducing tumor growth in a xenograft mouse model of human H1975 cells [49]. Combination of the two drugs almost completely abrogated tumor growth, without causing toxic effects to mice. This promising outcome in part encouraged progression of Omomyc into human clinical trials [12], which would be a significant milestone for a direct c-Myc inhibitor.

Since Omomyc is a mutant mini-protein of c-Myc, it has been speculated that, it may have issues relating to low proteolytic stability, potentially limiting its potential as a therapeutic. This hypothesis was challenged with Omomyc shown to persist for around 70 h in plasma [48, 49], although recent work has suggested that plasma concentrations rapidly decline after intravenous administration to healthy mice and that Omomyc is poorly distributed into tissues [104]. Formulation methods or synthetic modifications may therefore be required for therapeutic success. Interestingly, the size of the Omomyc protein makes it relatively accessible to chemical synthesis [155, 156], allowing for a variety of chemical modifications to potentially be tested.

## Other proteins and polypeptides that target c-Myc

### Max bHLHZip (Max*)

**Fig. 4:**
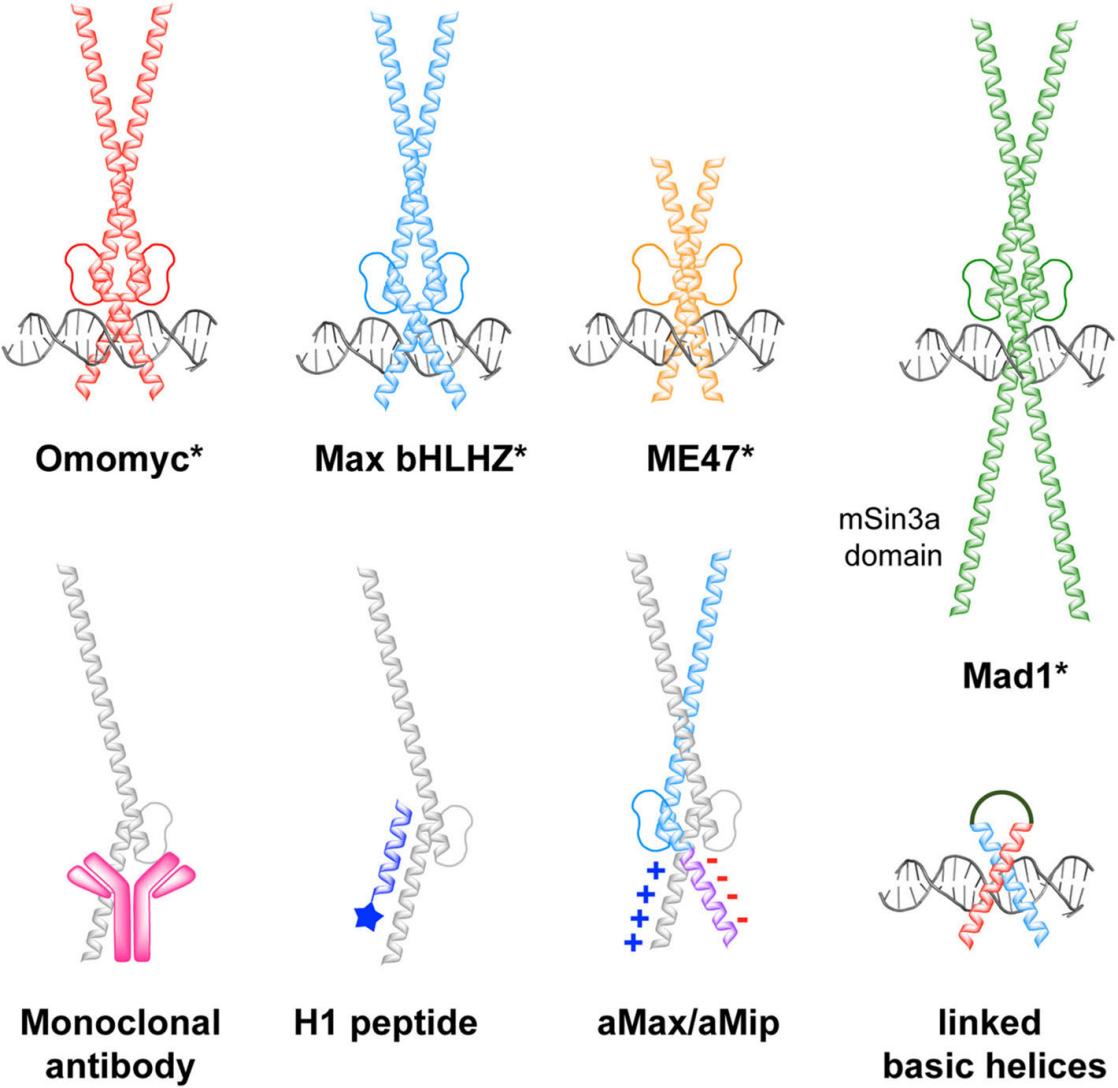
Overview of peptides and proteins that inhibit c-Myc, including E-box binding inhibitors (Omomyc, Max, ME47, Mxd and linked basic helices), inhibitors of c-Myc/Max binding (Omomyc, Max, Mxd, Monoclonal antibody, H1 peptide, a Max/aMip) and a c-Myc degradation promoter (Omomyc). Proteins marked with (*) are shown in their dimeric form [5, 99, 106, 107, 111, 112, 118, 120, 123]

Before recombinant Omomyc was reported to be active in cells, an 83-residue mini-protein featuring the bHLHZip domain of Max (Max*) was described as being cell permeable and a promising c-Myc transcriptional inhibitor in vitro, supposedly by forming Max* homodimers that compete for E-box binding sites (Fig. 4) [5]. Max* features a highly positively charged nuclear localization sequence at the DNA-binding basic region (KRAHHNALERKRR) and was shown to act as a PTD, entering HeLa cells via a endocytic pathway, partially escaping endosomes and translocating to the nucleus**.** Incubation with Max* reduced HeLa cell metabolism and proliferation, as well as repressing the expression of c-Myc activated genes. Although much less validated as a therapeutic agent than Omomyc, Max* has the advantage over Omomyc of not interacting with Miz-1 and thus not repressing expression of negative cell cycle regulators (e.g. CDKN2B and CDKN1A).

### Mad

The mini-protein, Mad, was recently reported to inhibit c-Myc and is based on the N-terminal 146 residues of the c-Myc-antagonist Mxd1, featuring its bHLH domain but also the Sin3a region that is capable of binding the epigenetic mSin3a repressor [106]. Additionally, a serine-to alanine mutation at position 145 avoids degradative ubiquitination, rendering the variant more stable than endogenous Mxd1. Mad was shown to be cell permeable and to reach the nucleus in HCT116 cells. In fluorescence polarization assays, Mad exhibited similar E-box binding activity as Omomyc. It was able to bind Max but not c-Myc in cells and interacted with nucleolar upstream binding factor (UBF) affecting rRNA synthesis. Remarkably, Mad was a ten-fold more potent inhibitor of cell proliferation than Omomyc in two different cancer cell lines, while ineffective in cells with low c-Myc expression. This greater potency was attributed to lack of Miz-1 activation by Mad, higher binding affinity for Max, or formation of c-Myc-repressing Mad/Mlx heterodimerization. These results suggested that Mad may be a more efficacious c-Myc inhibitor than Omomyc, but this is yet to be verified in animal models.

### ME47

MAXE47 (ME47) is a designed hybrid protein of the basic region of Max and the HLH region of another transcription factor, E47 (Fig. 4) [107–110]. ME47 was designed to homodimerize via its HLH domain and bind E-box DNA, allowing it to act a c-Myc inhibitor by competing with the c-Myc/Max dimer for DNA-sites in c-Myc target genes. An X-ray crystal structure of the dimer supported a homodimerization and E-box-binding mechanism [110]. ME47 showed high affinity for the canonical E-box in a FP assay and strongly competed with Max bHLHZip for E-box binding sites in yeast one-hybrid assays. Furthermore, transgenic expression of ME47 in breast cancer cells reduced cell proliferation to a comparable extent as Omomyc and inhibited tumor growth in a MDA-MB-231 breast cancer mouse xenograft model [109] . The smaller of size of ME47 relative to Omomyc (66 amino acids vs. 91 residues of Max or Omomyc), demonstrates that smaller HLH proteins can also block DNA binding sites. Unlike Omomyc, ME47 does not interact with other c-Myc/Max/Mxd network proteins making ME47 a valuable selective E-box tool that further validates inhibition of c-Myc binding to E-box DNA as a viable strategy for blocking abnormal c-Myc transcription. The cell permeability of ME47 and therapeutic potential as a protein drug candidate are yet to be reported.

### A monoclonal antibody to inhibit c-Myc

A c-Myc monoclonal antibody was isolated by immunising mice with the basic region of the c-Myc peptide (Fig. 4) [111]. The antibody recognized overexpressed, and endogenously expressed, c-Myc in cells and bound to the c-Myc epitope with high affinity (EC_50_ 40 nM). Further, the antibody inhibited c-Myc/Max binding to DNA, making it a promising starting point for further anti-c-Myc monoclonal antibody development. A key challenge is to find a way to deliver such antibodies into cells and furthermore to localize in the nucleus.

### Helix 1 c-Myc mimetic peptide

Early studies identified a short 14-amino acid c-Myc H1 peptide, (NELKRAFAALRDQI known as H1-S6A,F8A or H1) that was able to inhibit c-Myc/Max binding to DNA in gel mobility shift assays (Fig. 4) [112]. The H1 peptide contained S6A and F8A mutations versus the native c-Myc H1 sequence in order to increase *α*-helicity and heterodimeric binding with c-Myc. In addition to inhibiting c-Myc-Max dimerization [157], H1 peptide likely disrupts formation of the c-Myc/Max/Miz-1 repressive complex due to overlapping binding sites of H1 and Miz-1; an interaction important for normal cellular function and also suggested to be involved in oncogenic transformation [46, 156].

H1 cannot permeate membranes, so efforts have been made to deliver this epitope to the cell nucleus. Conjugation of H1 to 16-mer Antennapedia cell penetrating peptide vector (penetratin) [157] resulted in a new derivative, Int-H1, that could permeate and decrease the survival of MCF-7 breast cancer cells at low micromolar concentrations [113]. Without the two Ala substitutions, the peptide was inactive in cells and unable to inhibit c-Myc-Max interaction as verified by co-immunoprecipitation experiments. A retro-inversion analogue of Int-H1 designed to resist proteolysis was also shown to inhibit cancer cell proliferation [154, 157, 158].

The H1 peptide was conjugated to a thermally responsive biopolymer, Elastin-Like Polypeptide (ELP), that forms reversible aggregates at certain transition temperatures, so that peptide accumulation could be directed to where mild hypothermia was externally induced [114–116]. This construct was also fused to a series of cell penetrating peptides, such as penetratin, Tat and Bac. The Bac-ELP-H1 conjugate showed higher nuclear translocation and reduction of MCF-7 cancer cell proliferation. Treatment of breast tumors with Bac-ELP-H1 (but not controls lacking H1 peptide) and localized infrared illumination (up to 42 °C hyperthermia) led to significant reduction in tumor volume (~ 70%) in a mouse xenograft model. This strategy was also applied to enhance delivery of the c-Myc inhibitor via focused hyperthermia to brain tumors in rats with intracerebral gliomas.

A different nuclear delivery strategy, involving H1 conjugated to a cell-penetrating and nuclear translocating NrTP1 sequence (YKQSHKKGGKKGSG, epitope from a rattlesnake venom protein) and attached to a water soluble and lysosomally-cleavable HMPA (*N*-(2-hydroxypropyl)-methacrylamide) biopolymer, prolonged blood circulation and increased accumulation in tumors [159]. The drug-polymer construct showed improved antitumor efficacy in a HeLa xenograft mice model, inhibiting tumor growth by 77% compared with 28% by H1 alone. The same group had previously demonstrated that sequential administration of a HMPA-docetaxel conjugate (docetaxel is an antimitotic agent that weakens the nuclear envelope) followed by a HMPA-H1-conjugate also reduced tumor growth in similar mouse model.

A new Peptide Nuclear Delivery Device (PNDD) strategy applied to H1 peptide [160] involves a non-toxic truncated version (~ 42 kDa) of *Pseudomonas Exotoxin A*, a bacterial toxin that intrinsically translocates to the nucleus. This was coupled to H1 peptide, partially delivering it to the nucleus of MG63 cells. Remarkably, the PNDD-H1-fusion protein displayed 3 orders of magnitude greater potency than smaller CPP-H1-conjugates (cadherin, penetratin or TAT) in a c-Myc reporter assay in epidermoid carcinoma A431 cells, where high levels of c-Myc are present. Further validation was provided when PNDD-H1 decreased cell proliferation and induced substantial cell death in various tumor cell lines (but normal B-cells survived) using only 50–100 nM concentrations.

### Polypeptides with acidic extensions

Intending to generate dominant-negative derivatives of bHLHZip proteins, the basic region of Max was strategically replaced with an ‘acidic extension’ featuring a highly negatively charged sequence that electrostatically complemented the basic region of c-Myc (e.g. -**D**P**DEEEDDEEE**L**EE**L**E**D- substituted for -AD**KR**A**HH**NALE**RKRR**DHIKD-) (Fig. 4) [118]. The peptide was designed to bind to the basic region, via the acidic extension, in addition to the HLHZip region of c-Myc in order to extend the binding interface of the peptide and inhibit binding of the target to DNA. The resulting acidic Max (aMax) bound with high affinity to both the basic and leucine zipper region of c-Myc forming a more stable but transcriptionally inactive heterodimer relative to the native c-Myc/Max complex, thereby abolishing the binding of native c-Myc/Max to DNA. A similar strategy appended an acidic extension to the c-Myc-targeting peptide (Mip) selected from a genetic library to create aMip [119]. This was a superior inhibitor to Omomyc against c-Myc/Max/DNA complexation, according to gel shift assays. Guided by molecular dynamic simulations, aMip was later optimized by amino acid substitutions to substantially increase the stability of the c-Myc/aMip dimer (*T*_m_ increased from 46 °C to 64 °C) [120].

### Polypeptide mimics of the DNA-binding domain of c-Myc/Max

While most studies have focused on inhibiting c-Myc/Max dimerization, peptides have also been designed to bind directly to E-box DNA in order to block c-Myc/Max binding (Fig. 4, Table 1). Studies involving the yeast transcriptional activator GCN4, a bZIP protein, demonstrated that 26–34 residue peptides corresponding to the basic domain could be appended by a synthetic linker to assemble a forking helical homodimer capable of binding to DNA with high affinity [161–163]. Subsequent work showed that a synthetic covalently bonded c-Myc/Max complex could bind to E-box DNA, the approach of linking transcription factor basic domains was expanded to the bHLHZIP region c-Myc/Max interaction [121, 122]. In the case of bHLHZip dimers like c-Myc/Max, the dimerization and DNA-recognition domains are intervened by a loop, making the design of such DNA-binding peptide “tweezers” considerably more challenging. Nevertheless the c-Myc/Max dimer linkage was approached using a steroid-based scaffold to provide structural rigidity and to improve bioavailability, peptide stability and cellular uptake [122, 164–166]. However, problems such as non-specific DNA binding, incorrect peptide orientation upon DNA binding, and low *α*-helical stability, were identified*.* After structural modifications, a steroid-linked dimeric peptide was identified to bind to E-box DNA by inducing correct orientation of the basic peptides albeit with reduced affinity [123]. Despite these advances, these peptides have not been shown to inhibit c-Myc/Max binding to E-box DNA.

A homodimeric truncated version of Omomyc, depleted of the Zip domain but encompassing the bHLH sequence connected by a disulfide bridge, reduced proliferation of HCT116 cells comparable to the full length Omomyc [155]. This suggests that the coiled-coil region (or a significant part of it) may not be necessary for activity. However, the DNA binding properties of this compound were not reported nor whether the covalent dimer could be shortened to the basic region alone.

Until recently, the basic domains of the c-Myc/Max complex were thought to be unstructured when unbound to DNA, but new crystal structures and NMR analysis revealed that these regions of the apo c-Myc/Max dimer can also populate helical conformations, implying a conformational selection for DNA binding, rather than an induced fit mechanism upon c-Myc/Max/DNA complexation [167]. This suggests that the binding affinity of linked basic domain inhibitors for E-box DNA may be further improved via chemical stapling of the basic region, to impose the optimal conformation for DNA binding. This approach might also be beneficial for other c-Myc inhibitors that act on E-box sites such as Omomyc and Max*.

## Future peptide-based approaches to target c-Myc

### Blocking binding of c-Myc/Max to E-box DNA

While attention has traditionally focused on blocking the c-Myc/Max interaction, it is now recognized as important to focus on identifying the most effective inhibitors of c-Myc/Max/E-box ternary complex formation since this will ultimately lead to inhibition of c-Myc transcriptional activation. Discovery of inhibitory and antiproliferative Omomyc activity in vivo, which has also been shown to inhibit such formation, points to the potential of targeting the c-Myc/Max/E-box ternary complex [49]. Future work should focus on optimising the synthetic linker between the two basic domains in order to achieve the best orientation of the two basic domains. Studies should also explore chemical stapling of these basic region peptides to induce the bioactive conformation, which could help create inhibitory peptides with even greater potency.

### Selective targeting of c-Myc

Selectivity can be very difficult to achieve for peptides by rational design, since they can have many potential interaction partners in addition to the desired target [168, 169]. However, library screening approaches capable of considering multiple off-targets in addition to the target are being developed. Library-based identification of target selective peptides has already been employed for other systems, such as the oncogenic transcriptional regulator, Activator Protein-1 [169]. In that case polypeptides were derived to selectively bind to key Jun or Fos components with minimal crosstalk using a Competitive and Negative Design Initiative (CANDI). The CANDI approach works by explicitly considering off-target proteins during peptide library screening. Thus, library members that bind the off-target, or that promiscuously bind both target and off-target, are outcompeted by those that are highly target-selective. The CANDI approach could potentially be used to screen for polypeptides and/or optimize current c-Myc inhibitor peptides that selectively inhibit the c-Myc/Max interaction over other protein-protein interactions in the Myc/Max/Mxd network [169, 170]. Intracellular selection methods offer additional advantages, such as the selection of soluble, non-toxic polypeptides with significant levels of proteolytic stability [171]. It would be interesting to explore the biological effects of a CANDI-optimized Omomyc and explore whether it had greater potency in vivo.

### Therapeutics based on Omomyc

Omomyc has been shown to induce tumor regression in several cancer models, including some for which there are limited therapies, such as pancreatic cancer [43–45, 49, 100–104, 149]. Further studies are required to identify if Omomyc itself is a sufficient CPP to induce extensive tumor regression in patients. Work is also required to explore whether limitations in regards to proteolytic instability can be overcome [104]. Standard modification strategies could be explored in an attempt to improve stability, cellular uptake, or biodistribution of Omomyc or its derivatives, for example by *N*-methylation of the peptide backbone or the use of D-amino acids [144, 172].

If these limitations cannot be overcome, miniaturisation of Omomyc might provide an alternative route to potent therapeutics with better pharmacokinetic properties. For example, an understanding of the functional components of Omomyc may catalyse separate efforts based on the basic region for blocking E-box binding and the HLH or leucine zipper for sequestering Max away from c-Myc/Max complex formation. Indeed work has also been conducted to explore whether a stapled peptide of the leucine zipper of Omomyc could act as a suitable antagonist [173].

### Targeting c-Myc for degradation

Omomyc reduces c-Myc levels due to the proteasomal degradation of c-Myc [104]. Since Omomyc has induced tumor regression in multiple cancer models, promoting c-Myc degradation might be another viable therapeutic strategy (Fig. 3). PROteolysis-TArgeting Chimeras (PROTACs) are heterobifunctional small molecules that simultaneously bind a target and an E3 ubiquitin ligase, inducing the ubiquitination and subsequent degradation of the target by the proteasome [174]. A peptide PROTAC recently developed was able to recruit the E3 ubiquitin ligase Keap1 to degrade Tau. This finding opens the way for PROTAC, including peptide-based PROTACs, to be examined for targeting proteins such as c-Myc [60, 175]. There could be other therapeutic strategies to reduce the levels of functional c-Myc, such as inducing the formation of c-Myc aggregates using a peptide with an aggregating region [176]. Macrocyclic peptides were also recently reported to enhance c-Myc degradation by an unknown mechanism [177].

## Conclusions and future perspectives

Although there is no current therapy targeting c-Myc in the clinic, studies over the last two decades have provided great insights into problems that limit targeting of c-Myc with inhibitors [73]. New strategies have been identified for facilitating the development of potent small molecule inhibitors with favorable pharmacokinetic properties, although these are still not well advanced. Omomyc and related polypeptide inhibitors of c-Myc function can also reduce proliferation of cancer cells in vitro and tumors in vivo, demonstrating that casting the net beyond conventional small molecule inhibitors could be a viable alternative strategy towards treatment for a wide variety of c-Myc-dependent cancers. At the very least these studies have provided fundamental new mechanistic insights and clues that highlight promising approaches towards inhibiting c-Myc, such as preventing its binding to E-box DNA, which may pave the way to the development of the first effective treatment that targets c-Myc.

## Data Availability

All data and materials are included in this published article.

## Abbreviations

aMax: Acidic Max
aMIP: Acidic Myc-interfering peptide
AURKA: Aurora kinase A
BioID: Biotin identification protocol
bHLHZip: Basic-helix-loop-helix-leucine zipper domain
BRD4: Bromodomain protein
CANDI: Competitive and Negative Design Initiative
CD: Circular Dichroism
CDK4: Cyclin dependent kinase 4
CHIP: Chromatin immunoprecipitation
CPP: Cell penetrating peptide
c-Myc: Myelocytomatosis oncogene cellular homolog
EMSA: Electrophoretic Mobility Shift Assay
E-box: Enhancer box
FBW7: F-box and WD repeat domain containing 7
FP: Fluorescence Polarization
FPPa: Functional penetrating ‘Phylomer’ peptide
GCN4: General control non-repressible protein 5
HDAC: Histone deacetylase
HEK293T: Human Embryonic Kidney 293 T cancer cell line
HLH: Helix loop helix domain
ILC2s: Group 2 innate lymphoid cells
Mad/Mxd: Max-associated dimerization protein
Mga: MAX gene-associated protein
Max: Myc-associated factor X
Max*: Mini-protein featuring Max bHLHZip domain
MCF-7: Michigan Cancer Foundation-7 breast cancer cell line
Miz-1: Myc-interacting zinc finger protein-1
Mlx: Max-like protein X
Mnt: Max’s Next Tango
MONDO: bHLHZip heterodimerization partner for Mlx
Mxi1: Max-interacting protein 1
Omomyc: A 91-residue c-Myc dominant negative mini-Protein
p300: CBP-associated factor
P-TEFb: Positive transcription elongation factor b
PAF1: RNA polymerase II-associated factor 1 homologue
PEST: Domain rich in proline, glutamate, serine and threonine
PKD: Olycystic kidney disease
PROTACs: PROteolysis-TArgeting Chimeras
SKP2: S-Phase Kinase Associated Protein 2
SPR: Surface Plasmon Resonance
Sin3: Paired amphipathic helix protein
TIP48: ATP-binding protein
TRRAP: Transformation/transcription domain-associated protein
WDR5: WD repeat-containing protein 5
Zip: Leucine zipper domain
